# Arsenic Secondary Methylation Capacity Is Inversely Associated with Arsenic Exposure-Related Muscle Mass Reduction

**DOI:** 10.3390/ijerph18189730

**Published:** 2021-09-15

**Authors:** Md. Khalequzzaman Sarker, Selim Reza Tony, Abu Eabrahim Siddique, Md. Rezaul Karim, Nazmul Haque, Zohurul Islam, Md. Shofikul Islam, Moriom Khatun, Jahidul Islam, Shakhawoat Hossain, Zahangir Alam Saud, Hideki Miyataka, Daigo Sumi, Aaron Barchowsky, Seiichiro Himeno, Khaled Hossain

**Affiliations:** 1 Institute of Biological Sciences, University of Rajshahi, Rajshahi 6205, Bangladesh; drkazaldmc@gmail.com; 2 Department of Biochemistry and Molecular Biology, University of Rajshahi, Rajshahi 6205, Bangladesh; selimrezatony.789@gmail.com (S.R.T.); a.eabrahim@gmail.com (A.E.S.); nh676817@gmail.com (N.H.); zohurulislambmb@gmail.com (Z.I.); mmoriom25@gmail.com (M.K.); zahidulislam.akash333@gmail.com (J.I.); shossain@ru.ac.bd (S.H.); zasaud@ru.ac.bd (Z.A.S.); 3 Department of Applied Nutrition and Food Technology, Islamic University, Kushtia 7003, Bangladesh; mrkarimanft@gmail.com (M.R.K.); shofik.anft@gmail.com (M.S.I.); 4 Laboratory of Molecular Nutrition and Toxicology, Faculty of Pharmaceutical Sciences, Tokushima Bunri University, Tokushima 770-8514, Japan; hideki@ph.bunri-u.ac.jp (H.M.); sdaigo@ph.bunri-u.ac.jp (D.S.); himenos@ph.bunri-u.ac.jp (S.H.); 5 Department of Environmental and Occupational Health, University of Pittsburgh, Pittsburgh, PA 15261, USA; aab20@pitt.edu; 6 Division of Health Chemistry, School of Pharmacy, Showa University, Tokyo 142-8555, Japan

**Keywords:** arsenic, metabolites, muscle mass, insulin resistance, diabetes, cardiometabolic disease

## Abstract

Skeletal muscle mass reduction has been implicated in insulin resistance (IR) that promotes cardiometabolic diseases. We have previously reported that arsenic exposure increases IR concomitantly with the reduction of skeletal muscle mass among individuals exposed to arsenic. The arsenic methylation capacity is linked to the susceptibility to some arsenic exposure-related diseases. However, it remains unknown whether the arsenic methylation capacity affects the arsenic-induced reduction of muscle mass and elevation of IR. Therefore, this study examined the associations between the arsenic methylation status and skeletal muscle mass measures with regard to IR by recruiting 437 participants from low- and high-arsenic exposure areas in Bangladesh. The subjects’ skeletal muscle mass was estimated by their lean body mass (LBM) and serum creatinine levels. Subjects’ drinking water arsenic concentrations were positively associated with total urinary arsenic concentrations and the percentages of MMA, as well as inversely associated with the percentages of DMA and the secondary methylation index (SMI). Subjects’ LBM and serum creatinine levels were positively associated with the percentage of DMA and SMI, as well as inversely associated with the percentage of MMA. HOMA-IR showed an inverse association with SMI, with a confounding effect of sex. Our results suggest that reduced secondary methylation capacity is involved in the arsenic-induced skeletal muscle loss that may be implicated in arsenic-induced IR and cardiometabolic diseases.

## 1. Introduction

Due to its ubiquitous and poisonous nature, arsenic has posed a serious threat to human health worldwide. In some countries, particularly Bangladesh, arsenic poisoning has affected millions of people who mainly rely on groundwater heavily contaminated with arsenic. Although arsenic exposure-related mortality is mainly from malignant diseases, growing evidence suggests that cardiovascular disease is also a major cause of morbidity and mortality in arsenic-endemic areas [1,2,3,4,5]. In addition, our group and others have reported a link between arsenic exposure and diabetes mellitus [6,7,8,9,10,11]. We have recently found that the skeletal muscle mass, as assessed by serum creatinine and lean body mass (LBM) levels, was reduced in the Bangladeshi population exposed to arsenic and that the extents of the decrease in skeletal muscle mass were inversely correlated with the increases in the insulin resistance (IR) marker homeostasis model assessment of insulin resistance (HOMA-IR), suggesting that skeletal muscle may be a target of arsenic toxicity [7].

The reduction of skeletal muscle mass is a characteristic feature of aging and cardiometabolic diseases [12,13]. The decrease in skeletal muscle mass is also associated with pathogenic increases in the intramuscular fat (myosteatosis) [14,15,16], which leads to the promotion of IR [17,18]. Recently, experimental studies using animals and cultured cells have shown that arsenic affects the functions and regeneration of muscle [19,20,21]. However, less attention has been paid to the effects of arsenic exposure on the quantity and quality of the muscle in human studies. Our recent study was the first to demonstrate that arsenic exposure causes the loss of skeletal muscle mass in humans exposed to arsenic [7].

Many factors have been shown to influence the individual risk for arsenic-induced diseases [22,23,24,25,26,27]. The capacity of arsenic methylation is a well-known modifier of arsenic-induced skin lesions, diabetes, and metabolic syndromes [28,29,30,31]. Inorganic arsenic (iAs) ingested from water and foods is metabolized to methylated arsenic species by arsenic (+3 oxidation state) methyltransferase (AS3MT), mainly in the liver in humans [32,33]. AS3MT methylates iAs to monomethylarsonic acid (MMA) in the first step and then to dimethylarsinic acid (DMA) in the second step. Since MMA and DMA are readily excreted in the urine, the profile of urinary arsenic metabolites has been used in assessing the individual arsenic methylation status. The arsenic methylation capacity is expressed as a primary methylation index (PMI) (a ratio of MMA to iAs in the urine) and a secondary methylation index (SMI) (a ratio of DMA to MMA in the urine).

Methylation of iAs to organic metabolites mainly functions as detoxification and elimination pathways of arsenic. However, arsenic methylation also produces highly toxic and unstable intermediates, as well as methylated trivalent forms of arsenic such as MMAs^III^ and DMAs^III^ [33,34,35]. Thus, arsenic methylation has dichotomous roles in the detoxification and metabolic activation of arsenic. The individual capacity of arsenic methylation appears to be associated with the susceptibility to arsenic-induced diseases but in different ways depending on the types of disease. A meta-analysis of previous studies has shown that a higher percentage of MMA in the total urinary arsenic was associated with increased risks of several types of cancer and cardiovascular diseases (CVDs), and that a lower percentage of MMA and higher percentage DMA were associated with an increased risk of diabetes [36].

We have previously shown that arsenic exposure decreased muscle mass and increased IR [7]. However, it remains unclear whether the arsenic methylation capacity plays a modifying role in the arsenic-induced muscle mass loss and IR. Since muscle atrophy and dysfunctions have been increasingly recognized as significant risk factors for cardiometabolic diseases [16,37], it should be addressed whether the individual’s methylation capacity influences arsenic-induced muscle mass loss. Therefore, we investigated the associations between arsenic methylation capacity and muscle mass reduction in the subjects recruited from low- and high-arsenic exposure areas in Bangladesh. Since a majority of the subjects also participated in our previous study [7], in which HOMA-IR was measured, we also examined whether the arsenic methylation capacity is associated with arsenic-induced IR.

## 2. Materials and Methods

### 2.1. Ethical Permission

Institutional ethical permission was received from the Institute of Biological Sciences, University of Rajshahi (661/320/IAMEBBC/IBSc).

### 2.2. Study Areas

As with our previous studies [6,7], this study was conducted in the northwest region of Bangladesh, where high arsenic exposures are prevalent [38]. The locations of exposure areas were identified in consultation with a local health office and local health workers. After identifying the high-exposure areas, we visited each area with a physician who scanned villagers’ typical skin symptoms of arsenicosis. We found many villagers had hyperkeratosis, melanosis, and hard patches on their soles and palms, the characteristic arsenical skin lesions. The high-arsenic exposure villages that we selected for our current study included: Marua in the Jessore District; Dutpatila, Jajri, Vultie, and Kestopur in the Chuadanga District; and Kazirpara in the Rajshahi District. We also selected a low-arsenic exposure village named Chowkoli in the Naogaon District, where no apparent arsenic contamination of groundwater was recognized, as we described previously [39].

### 2.3. Study Subjects

We initially visited the 212 and 58 families from high- and low-arsenic exposure areas, respectively. All adult family members were asked to participate in our study and the individuals who volunteered for this study were requested to attend a specific location in each village for their primary enrollment. Physicians involved in our study carefully checked the arsenical skin lesions among the residents in high-exposure areas. A dermatologist further confirmed the subjects who had skin lesions. Only local adult (18–60 years old) residents living in their respective areas for a minimum of five years were recruited for this study. In response to our request, 347 individuals from 205 families in high-arsenic exposure areas and 98 participants from 56 families in low-arsenic exposure areas were primarily enrolled. We enrolled the residents to participate in our study irrespective of their skin lesions. We also set some other exclusion criteria that included: hepatotoxic or anti-hypertensive drug use; malaria or leishmaniasis; a history of hepatic, renal, or severe cardiac diseases; a recent history of surgical operation; pregnant and lactating mothers; and any history of drug addiction. Three (Hepatitis B = 1, stroke = 1, and pregnancy = 1) of 347 individuals from high-exposure areas and two (Hepatic disease = 1 and renal disease = 1) of 98 individuals from the low-exposure area were excluded from the study according to the exclusion criteria. An additional three participants from high-exposure areas who donated blood were also excluded after performing laboratory experiments, as their serum creatinine levels were above 1.20 mg/dL. Finally, 437 subjects (n = 341 from high-exposure areas and n = 96 from the low-exposure area) were eligible for this study. We collected written consent from each subject and committed to strictly maintaining the confidentiality of the subjects.

### 2.4. Questionnaire and Interview

A structured questionnaire-based interview was conducted for each study subject by the trained team members, as described elsewhere [40,41]. We obtained the major information through the questionnaire: source of water for drinking and household uses, water consumption history, smoking status, income, education, occupation, marital status, and physical complications (if any).

### 2.5. Collection of Water and Measurement of Arsenic

We described the water collection process and measurement of arsenic in our previous report [39]. Briefly, we collected the water samples from tube wells identified by the study participants as their primary source of drinking water. Before collecting the samples, the tube wells were pumped for 5 min and water was then collected in an acid-washed container. The water samples were immediately acidified with nitric acid and then shipped to the Laboratory of Molecular Nutrition and Toxicology, Tokushima Bunri University, Tokushima, Japan, for the measurement of arsenic. Arsenic concentrations in the samples were measured by an inductively coupled plasma mass spectrometer (ICP-MS) (Agilent 7700×, Tokyo, Japan). The accuracy of the measurement was verified by the concentrations of arsenic in “River water” (National Metrology Institute of Japan [NMIJ] CRM 7202-a number 347, National Institute of Advanced Industrial Science and Technology, Tokyo, Japan) as certified reference materials (CRM). Duplicate water samples and triplicate CRM samples were used in the measurement. The average (mean ± SD) arsenic concentration in the triplicate CRM samples was 1.161 ± 0.082 mg/L (certified value: 1.18 mg/L).

### 2.6. Collection of Urine and Measurement of Urinary Arsenic Species

We provided acid-washed, cleaned urine collection tubes to each study subject and requested to collect the mid-stream of the first void urine in the morning. The urine samples were kept in a portable cooler box, immediately transported to our laboratory, and stored at −20 °C. The samples were then shipped to the laboratory of our collaborator at Tokushima Bunri University, Tokushima, Japan.

Speciation analysis of the urinary arsenic species was performed using high-performance liquid chromatography (HPLC) in combination with inductively coupled plasma mass spectrometry (ICP-MS) according to the procedure reported by Suzuki et al. (2009) [42]. Urine samples were centrifuged at 3000 rpm and the supernatant was applied to the anion column (DionexIonPac AS22, 250 mm × 4.0 mm i.d., Dionex, CA, USA) for the separation of the arsenic species. The separation conditions were as follows: mobile phase, 20 mM NH_4_HCO_3_ at pH 10; flow rate, 1.2 mL/min; column temperature, 40 °C; and injection volume, 50 µL. The eluent was directly applied to HPLC (HP−4500, Agilent Technologies, Kanagawa, Japan) using yttrium as an internal standard for the element analysis. The quantification of each arsenic species was conducted using the standard solution of sodium meta-arsenite, disodium hydrogen arsenate (Wako Pure Chemical Industries, Osaka, Japan), MMA, DMA, and arsenobetaine (Torii Pharmaceutical Co., Yamanashi, Japan). The combined amount of iAs(III) and iAs(V) was used as the amount of iAs, and the sum of the amounts of iAs, MMA, and DMA was expressed as the amount of total arsenic (tAs) in urine. Percentages of iAs (%iAs), MMA (%MMA), and DMA (%DMA) were calculated by dividing the amounts of each arsenic species by that of urinary tAs.

### 2.7. Blood Collection and Measurement of Serum Creatinine

We requested the subjects to be fasted for 10–12 h at night. Fasting blood (3 mL) from each subject was collected and serum was prepared as described previously [43]. We measured serum creatinine levels using commercially available kits (CREATININE liquicolor; Human GmbH, Germany) and a biochemical analyzer (Humalyzer 3000, Human GmbH, Germany). The detection ranges of creatinine kits were 0.35–13 mg/dL.

### 2.8. Estimation of Lean Body Mass (LBM)

The Boer formula [44,45] was employed to calculate the subjects’ LBM as follows.

For males: LBM = (0.407 × W) + (0.267 × H) − 19.2

For females: LBM = (0.252 × W) + (0.473 × H) − 48.3

Here, “W” is the body weight in kilograms and “H” is the body height in centimeters.

### 2.9. Estimation of HOMA-IR

A large degree of the subjects in the current study (62%, n = 271) also participated in the previous study, in which HOMA-IR was determined [7]. We used these overlapping subjects (n = 271) for analyzing the associations of arsenic methylation capacity with HOMA-IR.

### 2.10. Statistical Analyses

The statistical analysis in this study was performed by SPSS software (version 21.0; SPSS, Chicago, IL, USA). Age, BMI, and income were categorized into tertiles with equal numbers of study subjects by frequency test. Characteristics (age, sex, BMI, income, occupation, education, smoking habit, and skin symptoms) and the distribution of the study subjects with regard to both the water arsenic levels and urinary arsenic profile measures were assessed by either independent sample *t*-test or one way ANOVA (F-test). The differences of muscle mass measures and HOMA-IR between low- and high-arsenic exposure areas were determined by an independent sample *t*-test. The associations of the concentrations of the urinary arsenic species and tAs with those of water arsenic were examined by the Spearman correlation coefficient test. The normality of estimation was verified by the Q-Q plot. A generalized linear regression model was used to estimate the associations of urinary tAs, percentages of arsenic species, SMI, and PMI with muscle mass measures and HOMA-IR. In this model, log-transformed values of urinary tAs, arsenic species, LBM, serum creatinine, and HOMA-IR were used to improve the quality of the estimation, and the model was adjusted for the age, sex, occupation, education, and smoking status of the subjects. The statistical significance of the *p*-value was defined as *p* < 0.05.

## 3. Results

### 3.1. Characteristics and Distribution of the Subjects Based on the Drinking Water Arsenic Levels and Urinary Arsenic Profiles

The characteristics and distribution of the study subjects regarding the water arsenic levels and urinary arsenic profiles are shown in Table 1. The older group (43–60 years old) showed lower percentages of iAs and higher PMI among the three age groups. The range of the BMI in the middle group was 19.11–22.2, indicating that the overall BMI of the subjects was low. The higher BMI groups had higher SMI than the lower BMI groups. Water arsenic levels and urinary arsenic profiles were found to be a little different among the groups of subjects distributed by the socioeconomic parameters (income, education, and occupation). No significant differences were found in water arsenic and urinary arsenic profiles, and neither in the PMI and SMI in the subjects of the three different income-groups. Although the water arsenic and total urinary arsenic levels, as well as the percentages of iAs, were not significantly different among the subjects distributed by education levels, the percentages of MMA were increased and percentages of DMA were decreased significantly with increasing education status. SMI was also decreased with increasing education status, however, the changes were marginally significant (*p* = 0.082). Farmers showed lower levels of urinary tAs than housewives and others. There were no smokers among the females. Male smokers had significantly higher percentages of DMA and lower percentages of iAs than non-smokers. The individuals with skin lesions showed significantly higher levels of drinking water arsenic, urinary tAs, and lower levels of SMI than the subjects without skin lesions. The lower SMI among the individuals with arsenic-induced skin lesions coincides with the results of other studies [30,31].

### 3.2. Correlations between Water Arsenic and Urinary Arsenic Species

**Figure 1:**
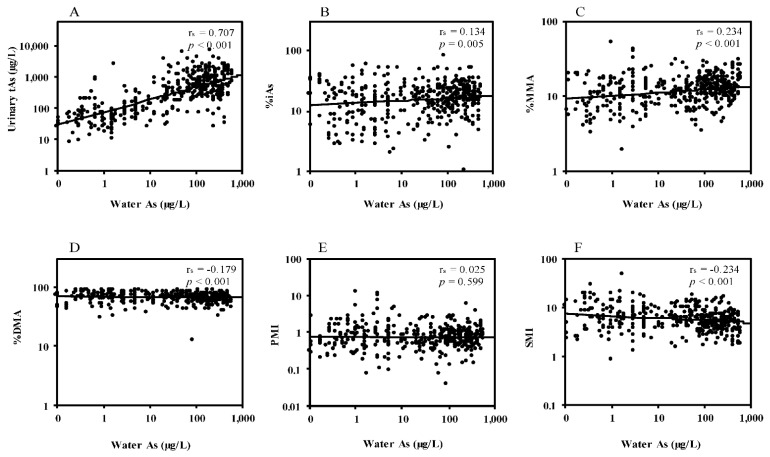
Association between water arsenic and urinary arsenic species. Associations be-tween water arsenic and total urinary arsenic (**A**), %iAs (**B**), %MMA (**C**), %DMA (**D**), PMI (**E**), and SMI (**F**). Results derived from Spearman correlation co efficient test.

Figure 1 shows the correlations between the arsenic levels in drinking water and the levels of urinary arsenic species. Water arsenic concentrations showed a strong correlation (r_s_ = 0.707, *p* < 0.001) with urinary tAs concentrations (Figure 1A). Water arsenic concentrations also showed positive correlations with the percentage of iAs (r_s_ = 0.134, *p* < 0.01) (Figure 1B) and percentage of MMA (r_s_ = 0.234, *p* < 0.001) (Figure 1C), and inverse correlations with the percentage of DMA (r_s_ = −0.179, *p* < 0.001) (Figure 1D) and SMI (r_s_ = −0.234, *p* < 0.001) (Figure 1F). PMI did not show a significant correlation with water arsenic concentrations (Figure 1E).

### 3.3. Effects of Arsenic Exposure on Muscle Mass Measures and HOMA-IR

Serum creatinine levels above reference values (0.6 to 1.2 mg/dL for males and 0.5 to 1.1 mg/dLfor females) are an indicator of kidney dysfunction. However, lower serum creatinine serves as a biomarker of skeletal muscle mass reduction [46]. To confirm whether arsenic exposure decreases muscle mass measures in the current study subjects (n = 437) as we observed in our previous study [7], we compared serum creatinine and LBM levels between the low- and high-arsenic exposure areas. As shown in Table 2, serum creatinine levels of the subjects in high-exposure areas were significantly lower than those of subjects in the low-exposure area in both sexes. The LBM level in the high-exposure areas was significantly lower than for those in the low-exposure area in females. These results, including the gender difference in LBM, are similar to the results in our previous study [7].

We also compared serum creatinine, LBM, and HOMA-IR levels between the low- and high-exposure areas among the overlapping subjects (n = 271) who participated in both the current and previous studies [7]. The HOMA-IR levels in the high-exposure areas were about two times higher than those in the low-exposure area, both in males and females (Table 2), similar to the previous study [7]. In addition, the results of the muscle mass measures were similar between the overlapping subjects (n = 271) and the subjects (n = 437) of our current study. Thus, our previous findings that arsenic exposure decreases muscle mass measures and increases IR were reproduced in the overlapping subjects. Furthermore, these results suggest that the study subjects were not selected with bias.

### 3.4. Associations between Urinary Arsenic Species and Muscle Mass Measures

The associations between the levels of urinary arsenic species and muscle mass measures were analyzed using linear regression analyses among the whole subjects (n = 437) (Table 3). We considered age, smoking, education, and occupation as possible confounders in regression analyses because these demographic and socioeconomic variables showed significant effects on the urinary arsenic species (Table 1). Additionally, we considered sex as a possible confounder as there is a baseline difference in the LBM levels between males and females [47,48]. In the linear regression, we found that urinary tAs, the percentage of MMA, and the percentage of iAs were inversely associated, while the percentage of DMA and SMI were positively associated, with LBM and serum creatinine levels after adjusting for the covariates. No significant associations were found between PMI and muscle mass measures. Among the demographic variables, sex was a significant confounder on the associations of urinary arsenic species with LBM. In sex-stratified analyses, the beta values for the associations of the percentage of MMA and SMI with both the LBM and serum creatinine levels in females were slightly higher than those in male subjects (Table 3).

### 3.5. Associations between Urinary Arsenic Species and HOMA-IR

We examined the associations of urinary tAs and arsenic species with HOMA-IR among the overlapping subjects (n = 271) through linear regression analysis (Table 4). We found that urinary tAs, the percentage of MMA, and the percentage of iAs were positively associated, while the percentage of DMA and SMI were inversely associated, with HOMA-IR after adjusting for covariates. PMI did not show any significant association with HOMA-IR. Since sex showed a significant confounding effect on the associations, we next performed sex-stratified analyses. The urinary tAs had significant positive associations with HOMA-IR in both sexes, confirming the enhancing effects of arsenic exposure on HOMA-IR as shown in Table 2 and in our previous study [7]. HOMA-IR showed a significant inverse association with SMI in females but the significance was marginal (*p* = 0.067) in males.

## 4. Discussion

To our knowledge, this study is the first to show the associations between the profile of urinary arsenic species and skeletal muscle mass, as well as their relations to IR, among human populations exposed to arsenic via drinking water.

In this study, the subjects’ arsenic exposure levels were assessed by the arsenic concentrations in drinking water and urine. A strong correlation between drinking water arsenic and urinary tAs concentrations (Figure 1A) indicates that our study participants were continuously exposed to arsenic from drinking water, the main route of arsenic exposure in Bangladesh. As in our previous study [7], the lower muscle mass measures and higher HOMA-IR in the high-arsenic exposure group relative to the low-exposure group were also confirmed in the current study subjects (Table 2). The urinary tAs levels showed an inverse association with serum creatinine levels (Table 3) and a positive association with HOMA-IR (Table 4), which is similar to our previous study that used hair and nail arsenic levels as the individual’s exposure marker [7]. Thus, the similar tendencies in the results across the different exposure markers in the two studies provide more definitive evidence for the effects of arsenic exposure on IR via the reduction of muscle mass.

The overall profile of the urinary arsenic species in this study (Table 1), such as the average levels of the MMA percentage (12.87%) and DMA percentage (68.77%); increased MMA percentage and decreased DMA percentage; SMI with increasing urinary tAs levels; and higher MMA percentage and lower SMI in the subjects with arsenic-induced skin lesions compared to the subjects without skin lesions are in good accordance with the previous studies conducted in the other high-arsenic exposure areas in Bangladesh, Taiwan, and China [23,24,27,30,31], as well as the results of the meta-analysis of urinary arsenic metabolites [36,49].

We found that muscle mass measures were inversely associated with the urinary MMA percentage and positively associated with DMA percentage and SMI (Table 3). Since the muscle mass measures were lowered by arsenic exposure (Table 2), these results suggest that the lower capacity of secondary arsenic methylation is associated with the lower skeletal muscle mass. The inverse association of SMI with HOMA-IR (Table 4) suggests that the lower capacity of the secondary arsenic methylation is also associated with increased HOMA-IR (Table 4). Thus, the lower capacity of the secondary arsenic methylation contributes to the increased susceptibility to the arsenic-induced reduction of skeletal muscle mass and IR. The data confirm the hypothesis that arsenic methylation is protective and suggest that methylation enzymes may have unknown benefits to muscle maintenance and metabolism.

Although the associations of MMA percentage, DMA percentage, and SMI with muscle mass measures were observed similarly in males and females (Table 3), the associations with HOMA-IR were more evident in females (Table 4). The more pronounced elevation of HOMA-IR associated with the muscle mass reduction more in females than males was also found in our previous study [7], confirming the higher sensitivity of females than males to the muscle reduction-related development of IR among the arsenic-exposed population. However, additional studies are needed to precisely explain the reason for the gender difference in the susceptibility to IR.

The reduced muscle mass quantity and quality have recently been recognized as crucial factors for increasing IR, promoting various cardiometabolic diseases [16,37,50]. Skeletal muscle accounts for a large part of insulin-mediated glucose uptake that contributes to a rapid decrease in elevated postprandial blood sugar. Several studies in humans have demonstrated that the low levels of skeletal muscle mass, as assessed by serum creatinine, were positively associated with the future risk of diabetes [51,52,53,54,55]. Additionally, it has been reported that the loss of muscle quantity increases intramuscular infiltration of lipids (myosteatosis), further contributing to IR development [17,18,50]. IR is not only the underlying mechanism of type 2 diabetes but also involved in the pathogenesis of cardiometabolic diseases, including hypertension. It promotes atherosclerosis, endothelial dysfunction, dyslipidemia, obesity, and imbalances between the production of vasodilators and vasoconstrictors [56,57]. Previous studies suggested that impaired arsenic methylation capacity is associated with several forms of cardiometabolic diseases such as hypertension, triglyceridemia, cholesterolemia, atherosclerosis, diabetes, and IR [24,28,58,59,60]. As an advancement of the previous findings, our results suggest that the lower capacity for secondary arsenic methylation with increasing exposure to arsenic could increase the susceptibility to muscle loss-associated IR, leading to elevated risks for cardiometabolic diseases in the individuals exposed to arsenic.

Only a limited number of human studies have examined the association of arsenic methylation capacity and muscle mass measures. Gribble et al. (2013) examined the relationship between the urinary arsenic species and body composition profiles, including fat-free mass in the Strong Heart Study in the US [61]. They reported that lower MMA percentage and higher DMA percentage are associated with higher fat-free mass. Recently, Abuawad et al. (2021) reported that SMI is positively associated with LBM among the participants in the cohort studies in Bangladesh (Folic Acid and Creatine Trial (FACT) and folate and oxidative stress (FOX) studies) [62]. Thus, our results are consistent with the results of these previous and ongoing studies.

In contrast, the inverse association between SMI and IR observed in our study is not consistent with previous studies [36,59,63,64,65]. These previous studies showed that lower MMA percentage or higher DMA percentage and SMI were associated with diabetes. One possible reason for the discrepancies is the differences in the subjects’ arsenic exposure levels. In the arsenic exposure areas of this study, the residents are currently exposed to moderate to high-levels of arsenic via drinking ground water. The maximum concentration of arsenic in drinking water exceeded 1000 µg/L. Using the samples collected from the residents of these high-exposure areas, we previously reported a link between arsenic exposure and hyperglycemia [6,7]. In contrast, most of the previous studies that examined the relationship between arsenic methylation capacity and diabetes have been conducted in the general population [64,65] or in the areas with low to moderate-level arsenic exposure [59,63]. For example, a study conducted in the arsenic-contaminated areas in Mexico showed an inverse association of HOMA-IR with arsenic concentrations in the water and urine [63]. However, the geometric mean values of iAs in the water and urinary tAs in their study were 24.4 and 24.7 µg/L, respectively, which were much lower than our data (geometric mean: 58.84 for water arsenic and 445.25 for urinary tAs) in the high-arsenic exposure areas.

Another possible reason for the discrepancies between our study and others may be related to the effects of obesity. Several studies have shown that BMI was associated inversely with MMA percentage and positively with DMA percentage [61,66,67]. Notably, 40–50% of the participants showed BMI values over 30 in the studies conducted in the US [61,67] and the average BMI of the participants in Lindberg’s study conducted in central Europe was 27 [66]. In contrast, the average BMI in our study participants was approximately 21 and only 1.6% of the participants showed a BMI over 30. Since obesity is the major risk factor for diabetes, it could be presumed that the higher SMI related to obesity confounded the relationship between arsenic methylation capacity and diabetes in the studies in the US and Europe.

Thus, our results demonstrated the inverse association of SMI with HOMA-IR among the least obese population exposed to moderate to high-level arsenic, whereas the other studies showed positive associations with SMI and diabetes among the populations with high obesity rates and low to moderate-level arsenic exposure. Since the high-level arsenic exposure itself may reduce SMI, as shown in Figure 1 and other studies [68,69], the lower SMI associated with the lower muscle mass measures might reflect the moderate to high-level arsenic exposure in our study subjects. However, lowered SMI has also been observed with many arsenic-related diseases, such as skin lesions, cancers, and CVDs, among the populations exposed to high levels of arsenic [23,24,30,31,70] and it seems reasonable that the lowered SMI is also associated with an increased risk of arsenic-related skeletal muscle loss and the resultant IR. Experimental studies support the findings of our study. Exposure to arsenic in vivo and in vitro caused impaired functions and regeneration capacity of the muscle [19,20,21,71]. Exposure to arsenic causes an impairment of muscle mitochondrial morphology and functions in mice [19]. These pathological changes caused by arsenic are associated with diminished muscle maintenance, force production, and regenerative potential. A recent study also suggested that inorganic arsenic exposure decreases muscle mass and enhances denervation-induced muscle atrophy in mice [72]. MMA is more toxic to cells at a lower concentration than inorganic arsenic such as arsenite [12,71]. It has been reported that trivalent MMA decreases the expression and nuclear localization of transcription factors involved in myogenesis [73]. Thus the increased production of MMA, because of the disruption of the secondary methyaltion capacity, may inhibit the myogenesis more efficiently than inorganic arsenic. Inhibition of myogeneis, i.e., the reduction of muscle mass, ultimately induces IR.

Notably, many animal studies have shown that exposure to arsenic in utero and in the postpartum period increased HOMA-IR in the offspring [74,75,76,77,78]. Considering the subjects in this study were relatively young (average age was approximately 37 years) and the arsenic contamination of tube-well water was first reported in Bangladesh about 30 years ago, a part of our study participants may have undergone prenatal and early-life exposure to arsenic.

There are contradictory reports on the sex differences regarding the methylation capacity. Some reports showed that females have higher secondary methylation capacity than males [79,80,81,82,83], while others did not find any significant sex differences [70,84,85]. Our study did not find a significant difference in SMI levels between males and females (Table 1). It is also difficult to assess the sex differences in arsenic methylation capacity in our study since there was a difference (marginally significant difference, *p* = 0.061) (Table 1) in urinary tAs levels between males and females. Females had higher average levels of urinary tAs than males, despite males and females having similar arsenic levels in drinking water (Table 1). However, this may reflect a cultural difference, since during the day, males in rural Bangladesh typically drink water from sources near the agricultural fields where they work and which are usually distant from their homes. Females, on the other hand, who were predominantly housewives in this study, spend most of their time at home. Females drink water from the relatively constant sources that we measured and we only used the home drinking water as the common exposure for both males and females. Thus, females at home may have been more constantly exposed to a high exposure source.

There are several limitations to our study. First, because of the cross-sectional design of this study, the observed associations could not provide evidence of exact causal relationships. Second, a major limitation is that we used the calculated LBM and serum creatinine levels as surrogate end points for the actual body composition due to the limited clinical resources in the study area. LBM does not differentiate muscle mass and fat mass that would be much better assessed by computed tomography [16,86]. Complementing the calculated LBM estimates by measuring serum creatinine levels provided additional confidence for interpreting the data and indicating changes in muscle mass. There is a concern that serum creatinine levels are affected by kidney function. However, serum creatinine levels under stable renal function reflect skeletal muscle mass and muscle energy metabolism of creatine [87,88]. We excluded three subjects who had serum creatinine levels above the normal range to remove the possibility of kidney dysfunction. The socioeconomic demographics of our study subjects were very similar, which suggests that other factors that affect serum creatinine, such as dehydration, diet, and drugs, would be normalized. Third, we did not examine the nutritional status of folate and vitamin B_12_, which affect the arsenic methylation capacity [68,80,89]. Methylation of arsenic and biosynthesis of creatine, the precursor of creatinine, are linked to one-carbon metabolism through folate and vitamin B_12_-dependent reactions [89,90]. Thus, reduced arsenic methylation could reflect protein-energy wasting in skeletal muscle due to poor nutrition and lack of creatine maintenance of skeletal muscle metabolism [91]. However, in our previous study of this population, we found that food intake and food habits were highly homogenous [40]. Thus, it is assumed that even if there were some nutritional deficiencies, it might affect all associations with a similar magnitude. Despite the noted limitations, the current study advances the understanding of correlations between IR (HOMA-IR) and arsenic metabolism, since our previous study was based on total arsenic measures in hair, nails, and drinking water [7]. The associations of urinary arsenic metabolites with IR strengthen the pathophysiological significance of arsenic exposure in promoting metabolic disease. However, more definitive studies of muscle mass effects are warranted to fully realize the importance of the pathogenic mechanism in cardiometabolic diseases promoted by chronic arsenic exposure.

## 5. Conclusions

This study showed that increased exposure to arsenic via drinking water decreased the secondary methylation capacity. Decreased secondary methylation capacity was significantly associated with reduced skeletal muscle mass and elevated IR, suggesting that the lower arsenic methylation capacity may increase the risk of cardiometabolic diseases through impairment of the skeletal muscle. These results will advance the understanding of the individual’s susceptibility to cardiometabolic diseases among the population exposed to arsenic.

## Figures and Tables

**Table 1:** Distribution patterns and characteristics of study subjects based on water arsenic and urinary arsenic profiles.

Variables		[n, %]	Water As (µg/L)	Urinary tAs (µg/L)	%iAs	%MMA	%DMA	PMI	SMI
			Mean (SD)	Mean (SD)	Mean (SD)	Mean (SD)	Mean (SD)	Mean (SD)	Mean (SD)
	All	437	128.44 (165.87)	635.89 (950.03)	18.35 (10.74)	12.87 (5.81)	68.78 (12.32)	1.02 (1.19)	6.78 (4.46)
Sex ^b^	Female	207 (47.4)	127.08 (161.96)	727.32 (1122.23)	19.36 (11.87)	12.96 (6.93)	67.69 (13.77)	1.08 (1.55)	7.13 (5.4)
	Male	230 (52.6)	129.67 (169.65)	553.6 (755.73)	17.45 (9.55)	12.79 (4.57)	69.76 (10.79)	0.98 (0.72)	6.46 (3.37)
	*p*-value		0.871	0.061	0.067	0.769	0.082	0.384	0.127
Age, years ^a^	18−31	151 (34.5)	131.64(187.43)	586.3 (643.4)	19.57 (9.45)	12.84 (5.42)	67.59 (12.38)	0.84 (0.65)	6.85 (4.65)
	32−42	142 (32.5)	126.99 (147.59)	596.99 (766.9)	19.35 (11.73)	12.62 (5.6)	68.03 (12.38)	1 (1.26)	6.75 (4.9)
	43−60	144 (33)	126.51 (159.81)	726.25 (1313.54)	16.09 (10.72)	13.15 (6.4)	70.77 (12.05)	1.25 (1.48)	6.73 (3.76)
	*p*-value		0.958	0.378	0.008	0.747	0.058	0.01	0.97
BMI ^a^	13.67−19.10	146 (33.4)	129.24 (146.15)	525.79 (635.11)	19.9 (9.63)	13.11 (4.89)	66.99 (10.96)	0.84 (0.65)	6.07 (3.11)
	19.11−22.20	145 (33.2)	124.54 (147.96)	658.25 (913.85)	16.61 (9.27)	12.88 (5.22)	70.51 (10.96)	1.13 (1.19)	6.73 (3.93)
	22.22−36.70	146 (33.4)	131.52 (199)	723.78 (1208.58)	18.53 (12.78)	12.62 (7.09)	68.85 (14.52)	1.11 (1.53)	7.53 (5.8)
	*p*-value		0.936	0.193	0.032	0.767	0.051	0.07	0.019
Income, USD ^a^	9.33−22.12	142 (32.5)	137.3 (163.78)	590.4 (980.4)	18.5 (9.67)	12.93 (5.43)	68.58 (11.56)	0.96 (1.08)	6.58 (3.98)
	22.13−26.64	149 (34.1)	111.58 (142.7)	595.21 (943.88)	17.74 (11.45)	12.56 (6.22)	69.7 (12.62)	1.1 (1.35)	7.32 (5.52)
	56.67−83.30	146 (33.4)	135.48 (185.59)	725.42 (966.37)	18.88 (11.04)	13.19 (5.95)	67.93 (12.77)	1.02 (1.2)	6.45 (3.84)
	*p*-value		0.379	0.4	0.675	0.675	0.492	0.626	0.227
Education ^a^	No formal education	242 (55.4)	130.01 (166.65)	614.21 (1035.91)	17.43 (10.7)	12.34 (5.94)	70.23 (11.94)	1.09 (1.36)	7.17 (4.22)
	Primary	127 (29.1)	121.94 (151.52)	656.96 (882.17)	19.72 (10.68)	13.12 (5.05)	67.16 (12.49)	0.87 (0.61)	6.51 (5.22)
	Above	68 (15.6)	135.02 (189.3)	673.67 (738.79)	19.07 (10.84)	14.29 (6.43)	66.64 (12.83)	1.08 (1.34)	5.9 (3.49)
	*p*-value		0.851	0.863	0.128	0.042	0.022	0.217	0.082
Occupation ^a^	Housewife	195 (44.6)	120.95 (153.5)	720.2 (1132.63)	19.43 (12.02)	12. 91 (6.99)	67.66 (13.79)	1.08 (1.57)	7.18 (5.48)
	Farmer	180 (41.2)	117.95 (140.23)	492.76 (582.76)	17.56 (9.71)	12.67 (4.41)	69.77 (10.69)	0.94 (0.6)	6.45 (3.26)
	others ^c^	62 (14.2)	182.45 (246.04)	786.23 (1126.25)	17.25 (9.03)	13.34 (5.30)	69.42 (11.74)	1.11 (1.08)	6.48 (3.79)
	*p*-value		0.021	0.027	0.166	0.731	0.232	0.457	0.247
Smoking ^b^	No	340 (77.8)	130.21 (169.83)	618.69 (939.08)	19.2 (11.29)	13.05 (6.17)	67.75 (12.75)	1.01 (1.27)	6.73 (4.68)
	Yes	97 (22.2)	122.23 (151.83)	696.16 (990.09)	15.36 (7.89)	12.25 (4.26)	72.39 (9.92)	1.07 (0.85)	6.96 (3.58)
	*p*-value		0.676	0.479	<0.001	0.147	<0.001	0.701	0.645
Skin symptoms ^b^	No	216 (49.4)	74.35 (129.75)	376.15 (692.86)	18.26 (11.13)	12.6 (6.61)	69.14 (13.09)	1.14 (1.61)	7.40 (5.44)
	Yes	221 (50.6)	181.23 (182.7)	890.85 (1110.21)	18.62 (10.35)	13.23 (5.05)	68.15 (11.58)	0.92 (0.62)	6.14 (3.19)
	*p*-value		<0.001	<0.001	0.733	0.276	0.414	0.07	0.005

Data presented as mean ± SD. Abbreviations: n, number of study subjects; As, arsenic; BMI, body mass index; LBM, lean body mass; MMA, monomethylarsonic acid; DMA, dimethylarsinic acid; tAs, total arsenic; PMI, primary methylation index; and SMI, secondary methylation index. BMI was calculated as body weight (kg) divided by body height squared (m^2^). Urinary tAs = [MMA + DMA + iAs]; %iAs = [iAs/urinary tAs] × 100); %MMA = [MMA/urinary tAs] × 100; %DMA = [DMA/urinary tAs] × 100; PMI = [MMA/iAs]; SMI = [DMA/MMA]. ^a^ *p* and ^b^ *p*-values were calculated by one way ANOVA (F-test) and independent sample *t*-test, respectively. ^c^ Others included business, student, workers, doctor, carpenter, driver, tailors, rickshaw puller, security guard, and retired workers.

**Table 2:** Comparisons of water arsenic, muscle mass measures, and HOMA-IR of male and female subjects between the low- and high-arsenic exposure areas.

Variables	Area	Male		Female	
		n	Mean ± SD	n	Mean ± SD
Water As (µg/L)	Low	41	2.48 ± 3.4	55	2.84 ± 4.76
	High	189	157.25 ± 175.4 ^#^	152	172.04 ± 167.69 ^#^
Serum creatinine (mg/dL)	Low	41	0.96 ± 0.16	55	0.86 ± 0.18
	High	189	0.80 ± 0.18 ^#^	151	0.77 ± 0.16 ^#^
LBM (kg)	Low	41	43.98 ± 6.96	55	36.58 ± 5.7
	High	189	43.57 ± 6.15	152	33.13 ± 8.08 ^#^
The subjects with the HOMA-IR measurement (n = 271) ^$^					
Water As (µg/L)	Low	29	2.35 ± 3.35	39	2.88 ± 4.84
	High	105	162.9 ± 156.77 ^#^	98	186.74 ± 156.77 ^#^
Serum creatinine (mg/dL)	Low	29	0.99 ± 0.14	39	0.9 ± 0.15
	High	105	0.78 ± 0.16 ^#^	98	0.77 ± 0.13 ^#^
LBM (kg)	Low	29	43.42 ± 6.89	39	37.24 ± 5.93
	High	105	42.84± 7.47	98	31.29 ± 8.09 ^#^
HOMA-IR	Low	29	0.81 ± 0.16	39	0.91 ± 0.37
	High	105	1.57 ± 0.8 ^#^	98	2.35 ± 1.88 ^#^

^#^ Significantly different (*p* < 0.001) from the low-exposure area by an independent sample *t*-test. ^$^ The subjects with the HOMA-IR measurement participated also in the previous study in which HOMA-IR values were determined (Mondal et al., 2020).

**Table 3:** Associations between urinary arsenic metabolites and muscle mass measures through linear regression analysis.

Variables	All ^a^				Male ^b^				Female ^c^			
	LBM (kg)	*p*-Value	Serum Creatinine ^d^ (mg/dL)	*p*-Value	LBM (kg)	*p*-Value	Serum Creatinine ^e^ (mg/dL)	*p*-Value	LBM (kg)	*p*-Value	Serum Creatinine ^f^ (mg/dL)	*p*-Value
	β (95% CI)		β (95% CI)		β (95% CI)		β (95% CI)		β (95% CI)		β (95% CI)	
Urinary tAs (µg/L)	−0.03 (−0.044, −0.016)	<0.001	−0.042 (−0.057, −0.026)	<0.001	−0.012 (−0.028, 0.004)	0.149	−0.056 (−0.079, −0.032)	<0.001	−0.046 (−0.069, −0.024)	<0.001	−0.029 (−0.05, −0.009)	0.005
Sex	−0.107 (−0.129, −0.085)	<0.001										
%iAs	−0.049 (−0.08, −0.019)	0.002	−0.065 (−0.099, −0.031)	<0.001	−0.041 (−0.077, −0.006)	0.024	−0.088 (−0.142, −0.033)	0.002	−0.055 (−0.104, −0.005)	0.03	−0.050 (−0.094, −0.007)	0.024
Sex	−0.108 (−0.13, −0.086)	<0.001										
%MMA	−0.16 (−0.202, −0.118)	<0.001	−0.141 (−0.189, −0.092)	<0.001	−0.141 (−0.193, −0.089)	<0.001	−0.114 (−0.197, −0.031)	0.007	−0.173 (−0.239, −0.107)	<0.001	−0.155 (−0.213, −0.097)	<0.001
Sex	−0.113 (−0.134, −0.091)	<0.001										
%DMA	0.231 (0.135, 0.327)	<0.001	0.228 (0.12, 0.337)	<0.001	0.211 (0.088, 0.333)	0.001	0.327 (0.141, 0.513)	0.001	0.243 (0.096, 0.389)	0.001	0.177 (0.046, 0.308)	0.008
Sex	−0.106 (−0.128, −0.084)	<0.001										
PMI	−0.022 (−0.051, 0.006)	0.119	−0.001 (−0.033,- 0.03)	0.927	−0.017 (−0.05, 0.017)	0.327	0.033 (−0.018, 0.084)	0.205	−0.027 (−0.072, 0.018)	0.236	−0.023 (−0.062, 0.017)	0.26
Sex	−0.109 (−0.132, −0.087)	<0.001										
SMI	0.133 (0.099, 0.166)	<0.001	0.121 (0.082, 0.159)	<0.001	0.119 (0.077, 0.16)	<0.001	0.116 (0.05, 0.182)	0.001	0.142 (0.09, 0.194)	<0.001	0.122 (0.076, 0.168)	<0.001
Sex	−0.11 (−0.132, −0.089)	<0.001										

^a^ Adjusted for sex (male used as a referent), age, smoking habit (non-smoker used as a referent), education, and occupation. ^b^ Adjusted for age, smoking habit (non-smoker used as a referent), education, and occupation. ^c^ Adjusted for age, education, and occupation. Log_10_-transformed values were used. Only significant associations are shown. ^d–f^ Data were missing on serum creatinine for three, two, and one study subjects, respectively.

**Table 4:** Associations of urinary arsenic metabolites with HOMA-IR through linear regression analysis.

Variables	HOMA-IR					
	All ^a^	*p*-Value	Male ^b^	*p*-Value	Female ^c^	*p*-Value
	Unstandardized β (95% CI)		Unstandardized β (95% CI)		Unstandardized β (95% CI)	
Urinary tAs (µg/L)	0.212 (0.17, 0.254)	<0.001	0.2 (0.14, 0.261)	<0.001	0.219 (0.159, 0.279)	<0.001
Sex	0.091 (0.021, 0.162)	0.011				
%iAs	0.143 (0.035, 0.251)	0.009	0.132 (−0.012, 0.276)	0.073	0.145 (−0.016, 0.306)	0.078
Sex	0.107 (0.025, 0.19)	0.011				
%MMA	0.376 (0.223, 0.53)	<0.001	0.18 (−0.057, 0.416)	0.135	0.471 (0.263, 0.678)	<0.001
Sex	0.135 (0.057, 0.214)	0.001				
%DMA	−0.447 (−0.778, −0.117)	0.008	−0.534 (−1.079, 0.011)	0.055	−0.393 (−0.832, 0.046)	0.079
Sex	0.107 (0.024, 0.189)	0.011				
PMI	0.026 (−0.073, 0.126)	0.605	−0.061 (−0.203, 0.081)	0.395	0.082 (−0.059, 0.223)	0.254
Sex	0.132 (0.048, 0.215)	0.002				
SMI	−0.305 (−0.428, −0.183)	<0.001	−0.174 (−0.359, 0.012)	0.067	−0.369 (−0.539, −0.201)	<0.001
Sex	0.12 (0.041, 0.198)	0.003				

^a^ Adjusted for sex (male used as a referent), age, smoking habit (non-smoker used as a referent), education, and occupation. ^b^ Adjusted for age, smoking habit (non-smoker used as a referent), education, and occupation. ^c^ Adjusted for age, education, and occupation. Log_10_-transformed values were used.

## Data Availability

Raw data are not openly available but can be made available from the corresponding author upon reasonable request.
